# Niches and Interspecific Associations of Dominant Populations in Three Changed Stages of Natural Secondary Forests on Loess Plateau, P.R. China

**DOI:** 10.1038/s41598-017-06689-9

**Published:** 2017-07-26

**Authors:** Li Gu, Zhi-wen Gong, Wei-zhong Li

**Affiliations:** 1 College of Forestry, Northwest A&F University, Yangling, P. R. China; 2 College of Economics & Management, Northwest A&F University, Yangling, P. R. China

## Abstract

Ecological niches remain central to explaining community structure, and niche-based studies have helped us to better understand species interactions, distributions, coexistence and associated mechanisms. *Quercus wutaishansea* populations on Loess Plateau are currently expanding their dominance in natural secondary forest types. In the present paper, we focused on the dynamic changes of niches and interspecific association among different changed stages. The overall interspecies association of the three communities exhibited positive association for Variance Ratio (VR) >1; the value of χ^2^ significance test further revealed that the overall association had reached the level of significant association. Among the total of 45 species pairs composed of 10 dominant species, in the *Pinus tabulaeformis* forest, the ratio of positive and negative associations was below 1, which 19 pairs showing positive association, 25 pairs showing negative association, and 1 pair showing no association; in contrast, in another forests, the ratios of positive and negative associations were above 1. The timing and consequences of these associations may illuminate how interaction mechanisms such as competition and alleopathy, structure changes. Then differences of species features in niches and associations should receive greater attention when planning forest management and developing restoration strategies.

## Introduction

Ecological communities are structured by complex networks of organisms and species interactions^1^. A given plant population does not ﻿only﻿ exist in isolation but coexists in the corresponding habitat and in association with other plant populations in time and space^2^. The relationship between species includes the competition for limited resources and mutual promotions^3^, which also determine the community structure and dynamics, and is mainly manifested by niche and interspecific association^4^. Both niche and interspecific association reflect the degree of interspecific competition and community stability^5, 6^. Therefore, studies on the relationships are important for a thorough understanding of the community composition and function, as well as changes dynamics, and can provide a theoretical basis for forest management and natural vegetation restoration.

Niche refers to the interplay and interaction between species and the environment^7^. Changes in niches during community changes reflect interspecific competition for resources, and a stable coexistence requires niche differentiation among competitors^8^. Theoretical and applied researches on niches have become a focus of scholars worldwide^9–11^. For example, the quantification of niche parameters, such as niche breadth and niche overlap, which are considered to be the determinants of species diversity and community structure, which not only reflect the population’s ability to use resources and characterize its functional status, but also reveal the role of various populations during community development, as well as shedding light on community stability. However, most of these studies focus on plants in one community, and few studies have explicitly quantified the aforementioned niche parameters in different changed stages^9^.

The importance of the interspecific association in determining community processes has been debated for decades^12^, which is one of the most important quantitative and structural characteristics of communities. Interspecific association indicates the spatial distribution relationship and functional dependency^13^. According to the habitat selection and mutual attraction or repellence of co-occurring plant species, interspecific association can be divided into three categories: positive association, negative association, and unrelated association^14^. The measurement of interspecific association facilitates a comprehensive understanding of community structure, function, formation, maintenance and changes^15, 16^. In China, because of restrictions caused by fieldwork conditions and the development status of regional forestry, research on the interspecific association of trees mainly focuses on the scale of the population or community. The research on plant interspecific associations began in the late 1970s, with pioneering studies mainly focused on interspecific correlation determination and sampling techniques to develop the theory and application^17^. The significance of interspecific association reflects differences in community habitat and niche overlap and separation, and a stability configuration model for the grouping of ecological species within communities was proposed^18^. Community stability was found to be fluctuation, especially in the process of changes^19, 20^. Aside from these investigations, there are little investigations on the interspecific associations of dominant species during each changed stage of the secondary changes process.

Over a long period, many scholars carried out studies on the changes sequence of forest vegetation using different methods on the Loess Plateau. From the research on vegetation variation in the region, this vegetation changes firstly occurred in herb and shrub communities, and *Pinus tabulaeformis* was a pioneer species that easily occupies bare land. *Quercus wutaishansea* was a mesopyhtic tree tolerant of cold and drought; its seeding was strongly tolerant of light limitation, which made it more fit for growing under trees. Once the *Pinus tabulaeformis* forest forms, the environments became favorable for other relatively shade-tolerant species (i.e., *Quercus wutaishansea*). The gradual establishment of the latter bringed a transition from the *Pinus tabulaeformis* forest to the *Pinus* + *Quercus* mixed forest. In the most recent 50 years, *Pinus tabulaeformis* forests on shaded slopes and half-shaded slopes had been or were in development toward *Quercus wutaishansea* forests^11^. Some scholars believed that the three communities, i.e., the *Pinus tabulaeformis* forest, *Pinus* + *Quercus* mixed forest and *Quercus wutaishansea* forest, were in different stages of the same changed sequence on the Loess Plateau, and the *Quercus wutaishansea* forest was the climax community in this region, and the *Pinus tabulaeformis* forest was the subclimax community^3, 18^.

Species association was a characteristic of community quantity and structure, which provide the basis for understanding interspecies relationships, and it was conducive to a correct understanding of the diversity conservation^21–23^. To examine the importance of plant-plant interactions in changes more fully, we explored the main associations among species based on the plot (1 hectare) survey, and analysis the dynamic changes of ecological niches, interspecific association. Sufficient consideration should be given to the biological characteristics to ensure that species maintained a reasonable composition and keep the community stable during conservation, development and utilization planning.

## Materials and Methods

### Study Area

**Figure 1:**
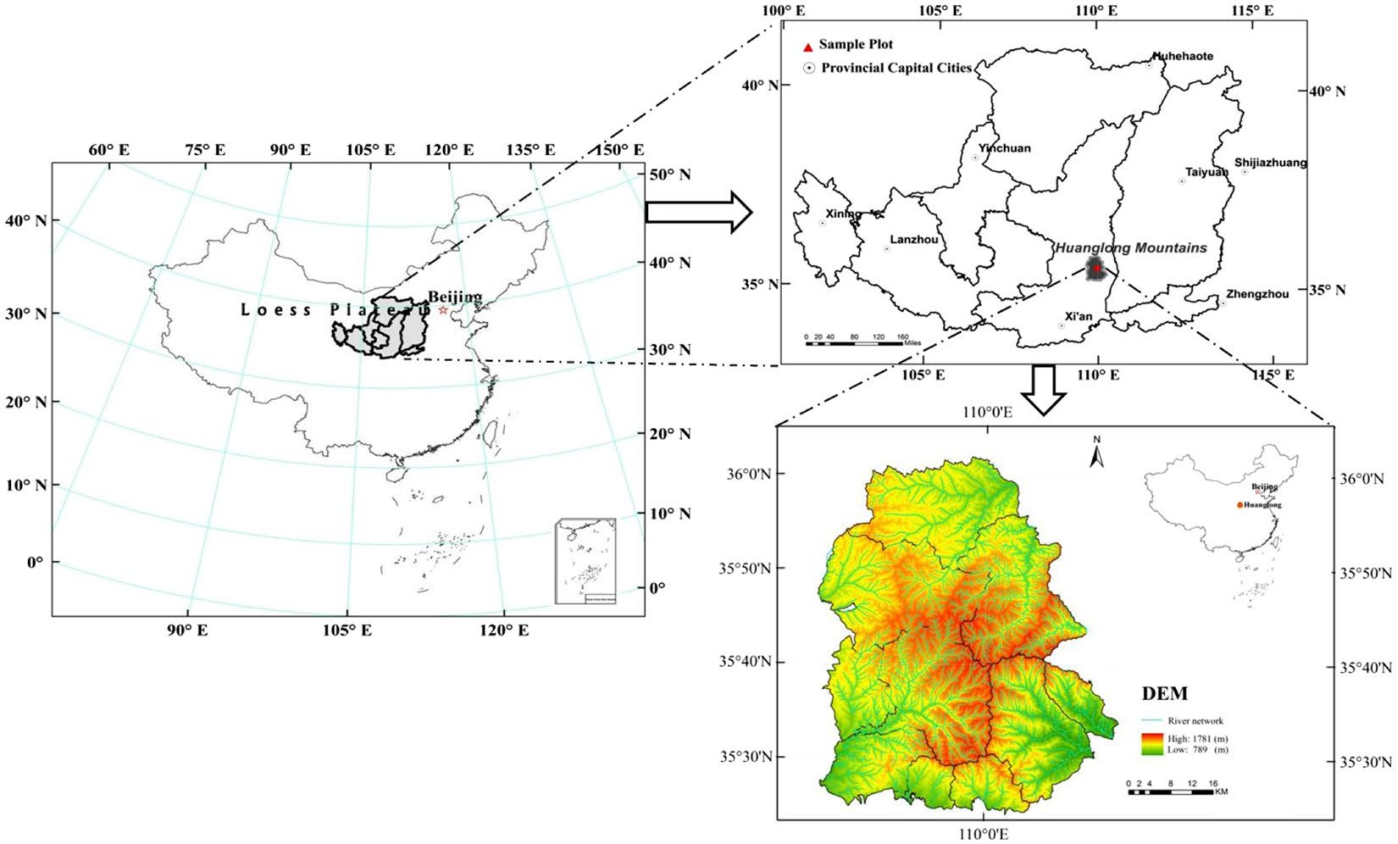
Location of the Loess Plateau and sample plot. The software of Arc GIS 10.2 was used to generate the maps, which was from the Digital Forestry Laboratory in College of Forestry, Northwest A&F University, and was bought in 2014.

The Loess Plateau is located in northeastern China and is one of China’s four large plateaus. This plateau is one of the birthplaces of ancient civilization in China and features the most concentrated distribution and largest areas of loess on Earth. Most importantly, the Loess Plateau shows some of the most serious areas of soil erosion and possesses one of the most vulnerable ecosystems in the world. The study area was located on the southeastern Loess Plateau in northern Shaanxi Province. Detailed surveys were conducted on Huanglong Mountains (Fig. 1). The entire forest zone shows extreme irregularity, a complicated geological structure, and a fractured landform, with the typical geographical structures of the Loess Plateau. The altitude ranges from 800 m to 2500 m, the annual average temperature is 10 °C, the frost-free period lasts 175 days, the annual precipitation is 611.8 mm (mostly between July and September), the annual evaporation capacity is 856.5 mm, and the general climate is within the warm temperate zone. The steep and hilly topography mainly consists of granite and gneiss. The mean slope is 35°, and the mean soil depth is 50 cm. The soil is classified as mountain brown earth.

The plants on the Loess Plateau consist of North China flora, with more than 580 plant species and 46 tree species in 29 genera and 22 families. The main tree species include *Pinus tabulaeformis*, *Betula platyphylla*, *Populus davidiana*, *Quercus wutaishansea*, *Acer ginnala*, *Pyrus betulaefolia, Toxicodendron vernicifluum, Crataeguscuneata*, *Populus simonii*, *Prunus davidiana, Juglans cathayensis* and *Betula albosinensis*.

### Field Measurements

According to our field investigation, *Pinus tabulaeformis* forest, although commonly seen, will for the most part be replaced by *Quercus wutaishansea* forest except on cliffs and ridge tops. Regenerated seedlings under the *Pinus tabulaeformis* forest on slopes were mainly *Quercus wutaishansea*. In some stands, *Quercus wutaishansea* occupies the second sub layer of the tree layer, revealing a notable tendency to replace *Pinus tabulaeformis*. Therefore, in this study, the research objects were the three typical community types in the late stage of the natural secondary changed series on the Loess Plateau: community I: *Pinus tabulaeformis* forest; community II: *Pinus* + *Quercus* mixed forest; and community III: *Quercus wutaishansea* forest.

Substituting space for time has been a widely used method for evaluating successional change^24^, which was initially used to distinguish changed stages and change in the woody vegetation. The method was inferred by assuming that time was the only difference and the time sequence represented vegetation through time. According to the space-time method, in Aug. 2013 and Aug. 2014, in accordance with the technical requirements on the arrangement of plots and the objective, three permanent sample plots were established, and each plot with an area of 1 hm^2^ (100 m × 100 m) in the natural secondary forest on Huanglong Mountain in Loess Plateau. Three plots with typical characteristics in good growth conditions in the late stage of a natural secondary changed series were arranged, i.e., Plot I was from community I: *Pinus tabulaeformis* forest; Plot II was from community II: *Pinus*+ *Quercus* mixed forests; Plot III was from community III: *Quercus wutaishansea* forest. Each community was sampled on both a sunny slope and a shaded slope (with a due east-due west line as the boundary, the slope at the southern boundary is the sunny slope and the slope at the northern boundary is the shaded slope). With an average tree age of more than 40 years and a canopy density of approximately 0.8 based on the adjacent gridding method, the plot was divided into 25 20 m × 20 m small plots by adopting high-precision TOPCON. In each small plot, the coordinates (x and y), diameter at breast height in 1.3 m (DBH), height (h), and the crown width of adult trees (DBH >3 cm) were measured. In addition, 5 2 × 2 m^2^ bush quadrats and 5 1 × 1 m^2^ plant quadrats (1 m × 1 m) were respectively arranged at the four corners and at the center of the subplot. The basic information of the sample was shown in Table 1.

**Table 1:** Basic information of the permanent samples.

Plot number	Area (ha)	Longitude (E)	Latitude (N)	Elevation (m)	Slop (°)	Aspect	Location	Average DBH (1.3 cm)	Average basal area (cm^2^)
Community I	1(100 × 100)	109° 46′	35° 4′	1351	31°	Southeast	middle	16.9	20.3217
Community II	1(100 × 100)	109° 58′	35° 37′	1430	26°	Northeast	middle	12.1	23.5662
Community III	1(100 × 100)	109° 57′	35° 31′	1505	29°	Northeast	middle	15.7	19.1445

### Data analysis

The importance value is a comprehensive quantitative indicator used to characterize the status and role of each species in the community, the larger the importance value of a species, the greater the dominance of the species in the plot, which was calculated with the following equation^25^:

1 $${\rm{IV}}=\frac{\frac{{{\rm{n}}}_{{\rm{i}}}}{{\sum }_{{\rm{i}}=1}^{{\rm{S}}}{{\rm{n}}}_{{\rm{i}}}}\,\ast \,100+\frac{{{\rm{a}}}_{{\rm{i}}}}{{\sum }_{{\rm{i}}=1}^{{\rm{S}}}{{\rm{a}}}_{{\rm{i}}}}\,\ast \,100+\frac{{{\rm{f}}}_{{\rm{i}}}}{{\sum }_{{\rm{i}}=1}^{{\rm{S}}}{{\rm{f}}}_{{\rm{i}}}}\,\ast \,100}{3}$$

where n_i_ is the number of individuals of the *i*th species, a_i_ is the basal area at the height of 1.3 m belonging to the *i*th species, f_i_ is the number of quadrats in which the *i*th species appeared, and *S* is the total number of species.

The niche breadth was calculated using the Shannon index^26^:

2 $${{\rm{B}}}_{{\rm{i}}}=-\sum _{{\rm{j}}=1}^{{\rm{r}}}{{\rm{p}}}_{{\rm{ij}}}\,\ast \,{{\rm{lgp}}}_{{\rm{ij}}}$$

where B_*i*_ is the niche breadth of the *i*th species, with the range as [1/r, 1], r is the total number of species, and P_ij_ is the proportion of the individuals of the *i*th species, which is associated with resource state j.

Niche overlap, of all species individually against other species combined^27^ and separately for each species pair, was calculated using Pianka’s index a_i_^28^:

3 $${{\rm{a}}}_{{\rm{i}}}={\sum }^{{{\rm{p}}}_{{{\rm{ijP}}}_{{\rm{ik}}}}}/{(\sum {{\rm{p}}}_{{\rm{ij}}}^{2}\sum {{\rm{p}}}_{{\rm{ik}}}^{2})}^{\frac{1}{2}}$$

where a_i_ is the niche overlap of the *i*th species, p_ij_ and p_ik_ are the proportions of the ith resource stage utilized by the species j and the other species combined (k), a_i_ ranges from zero (no overlap) to 1 (fully shared resource).

The variance ratio (VR) test^29^, which is based on species present or absence, and has been used to gain insight into the overall association among the different species, the VR was calculated as follows:

4 $${{\delta }_{T}}^{2}=\sum _{i=1}^{s}{P}_{i}(1-{P}_{i})$$

5 $${P}_{i}={n}_{i}/n$$

6 $${S}_{T}^{2}=(1/N)\sum _{j=1}^{n}{({T}_{j}-t)}^{2}$$

7 $$VR={S}_{T}^{2}/{\delta }_{T}^{2}$$

where S is the total number of species, n is the total number of quadrats, T_j_ is the number of individuals in the *j*th quadrats, *n*_i_ is the number of quadrats in which the *i*th species appeared, and t is the average number of species in the quadrats. The variance ratio = 1 under the assumption of independence, species show a positive association when VR > 1, and VR < 1 indicates that the species have a negative association.

Interspecific associations are comprised of positive association, negative association, and no association^30^. Positive association denotes that 2 species are likely to be found in the same location, and negative association denotes that species A is less likely to occur in the same community as species B. We used chi-square (*χ* ^2^) statistics based on a 2 × 2 contingency table to examine the interspecific associations^18^. The formula is as follows:

8 $${\chi }^{2}=\frac{{(|ad-bc|-0.5n)}^{2}n}{(a+b)(a+c)(b+d)(c+d)}$$

When χ﻿^2^ < 3.841, there is no interspecific association; when 3.84 <﻿χ﻿^2^ < 6.635, there are certain association between species; and when ﻿χ﻿^2^ > 6.635, there are significant associations between species. When ad > bc, the interspecific association is positive, and when ad < bc, the interspecific association is negative.

The association coefficient (AC)^20^ is used to quantify the interspecific association of each species pair, and the formula is as follows:

9 $${\rm{If}}\,{\rm{ad}}\ge {\rm{bc}}\quad {\rm{AC}}=\frac{({\rm{ad}}-{\rm{bc}})}{|({\rm{a}}+{\rm{b}})({\rm{b}}+{\rm{d}})|}$$

10 $${\rm{If}}\,{\rm{bc}} > ad,and\,d\ge {\rm{a}}\quad {\rm{AC}}=\frac{({\rm{ad}}-{\rm{bc}})}{|({\rm{a}}+{\rm{b}})({\rm{a}}+{\rm{c}})|}$$

11 $${\rm{If}}\,{\rm{bc}} > ad\,,\,and\,d < a\,AC=\frac{({\rm{ad}}-{\rm{bc}})}{|({\rm{b}}+{\rm{d}})({\rm{d}}+{\rm{c}})|}$$

The *AC* range is [−1, 1]; *AC* = 1 indicates the strongest positive interspecific association between species, and *AC* = −1 denotes that the species pair has the strongest negative interspecific association, while *AC* = 0 indicates that the species pair is completely independent.

From equations 8–11, Where n is the total number of quadrats, a is the number of quadrats with both A and B present, b indicates the number of quadrats with A only, c indicates the number of quadrats with B only, and d indicates the number of quadrats without A and B.

## Results

### Diameter Distributions

**Figure 2:**
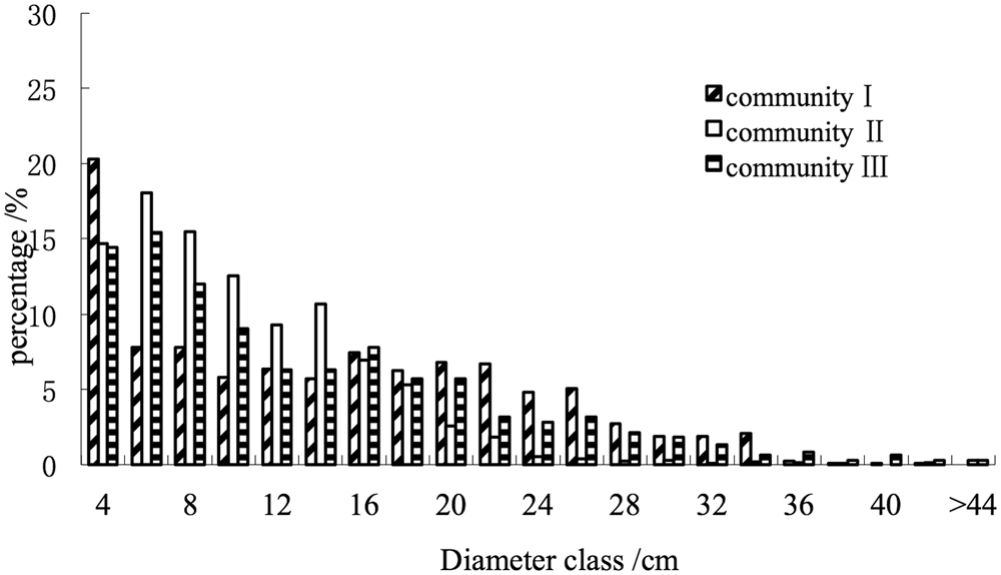
Dbh distributions of all species in the three 1-ha plots. X-axes display size classes in 4 cm intervals, y-axes display number percentage of individual trees.

All trees showed a reverse J-shaped distribution in both plots, with the number of individuals gradually decreasing with larger diameters (Fig. 2). The diameter structure was that the number of trees from diameter class 6 to diameter class 24 accounted for most of the stand. With the increase in the diameter class, the number of trees in each diameter class gradually decreased. When a certain diameter class was reached (Diameter class 6), the rate of decline remained the same. Although most trees were of medium and small diameter classes, trees with large diameters still existed.

### Importance Value and Niche Breadth

Among the permanent sample plots studied, the importance value of *Pinus tabulaeformis* was largest (56.14) in community I and was smallest (10.06) in community III; the importance values of *Quercus wutaishansea* varied more noticeably than other species, which varied from 16.17 in community I to 57.99 in community III (Table 2). The above 2 species were clearly dominant, for which the total importance values were highest, confirming them as the dominant species in the three communities.

**Table 2:** Importance value and niche breadth of dominant species in different communities (Importance value > 2).

Communities	Species	Ecological niche	Relative dominance	Relative frequency	Relative abundance	Importance value
CommunityI	*Pinus tabulaeformis*	1.36	0.86	0.22	0.61	56.14
	*Quercus wutaishansea*	1.30	0.07	0.20	0.22	16.17
	*Betula platyphylla*	1.19	0.03	0.17	0.08	9.41
	*Populus davidiana*	1.11	0.02	0.13	0.03	6.05
	*Swida macrophylla*	0.97	0.01	0.11	0.03	4.94
Community II	*Pinus tabulaeformis*	1.33	0.33	0.16	0.39	29.50
	*Quercus wutaishansea*	1.33	0.29	0.16	0.26	24.00
	*Lacquer tree*	1.32	0.30	0.16	0.24	23.27
	*Pyrus betulaefolia*	1.24	0.02	0.14	0.02	6.13
	*Populus davidiana*	1.12	0.03	0.11	0.03	5.80
	*Betula platyphylla*	1.36	0.02	0.12	0.03	5.61
Community III	*Quercus wutaishansea*	1.36	0.87	0.19	0.68	57.99
	*Pinus tabulaeformis*	1.21	0.03	0.17	0.10	10.06
	*Acer mono*	1.02	0.03	0.11	0.07	6.81
	*Swida macrophylla*	1.02	0.02	0.13	0.03	6.01
	*Populus davidiana*	1.14	0	0.11	0.06	5.67
	*Carya cathayensis*	0.58	0.01	0.05	0.02	2.72
	*Pyrus betulaefolia*	0.90	0.01	0.05	0.02	2.36
	*Lacquer tree*	0	0.01	0.05	0.01	2.13
	*Betula platyphylla*	0.89	0.01	0.05	0.01	2.12

The Shannon index was used to measure the niche breadth; in the process of forest changes, most mesophilous and shade species emerged and occupied a relatively large niche breadth. Before the establishment of *Quercus wutaishansea* (in community I), the species of *Pinus tabulaeformis* had the greatest niche breadth, i.e., 1.36, then followed by the establishment of *Quercus wutaishansea* and the pioneer species of *Populus davidiana* and *Betula platyphylla*, with the niche breadths of 1.30, 1.19 and 1.10, respectively; the niche breadth of xeric dwarf trees such as *Platycladus orientalis*, *Pyrus betulaefolia* and *Swida macrophylla* were relatively low. With the changes development, the niche breadth of *Quercus wutaishansea* was increased, from 1.30 to 1.36 (Table 2), but the value of *Pinus tabulaeformis* was decreased.

### Niche Overlap

With the changes of the dominant species, importance values, niche breadth and the niche overlap were changing. In the community I (the dominant species was *Pinus tabulaeformis*), the niche overlap between *Pinus tabulaeformis* and other species was within 0.597–1.455; in community III (*Quercus wutaishansea* as the majority), the niche overlap between *Quercus wutaishansea* and other species was within 0.231–1.459 (Table 3).

**Table 3:** Niche overlap of dominant species in different communities.

Communities	Species	1	2	3	4	5	6	7	8	9	10	11	12	13
Community I	2	0.96												
	3	0.96	0.73											
	4	0.69	0.63	0.47										
	5	0.87	0.63	0.89	0.36									
	6	1.04	0.93	0.64	0.54	0.19								
	7	0.74	0.60	0.52	0.30	0.11	0.20							
	8	0.60	0.73	0.17	0.45	0.05	0.20	0						
	9	1.40	1.40	0.98	0	0.35	0.33	0.33	0					
	10	0.88	0.82	0.52	1.13	0	1.00	0	0.71	0				
	11	0.88	0.82	0.52	1.13	0	1.00	0	0.71	1.00	0			
	12	1.46	1.01	0.69	0	0.26	0	0	0	0	0	0		
	13	0.88	1.19	0.35	0.38	0.26	0	0	0.71	0	0	0	0	
Community II	Species	1	2	3	4	5	6	7	8	9	10	11	12	13
	2	0.86												
	3	0.90	0.93											
	4	0.73	0.84	0.08										
	5	0.86	0.66	0.07	0.42									
	6	0.81	0.91	0.07	0.80	0.48								
	7	0.59	0.72	0.05	0.14	0.91	0.08							
	8	0.95	0.38	0.05	0.41	1.13	0.29	0.07						
	9	0.86	0.60	0.04	0.94	0.55	0.98	0	0.06					
	10	0.65	0.45	0.06	0.43	0.84	0	0	0.74	0				
	11	0.58	0.77	0.06	0.48	0.69	0.62	0	0	0	0			
	12	0.47	0.74	0.09	0.72	0.09	0.67	0	0	0	0.50	0		
	13	1.01	1.09	0.12	0.58	0.38	0.67	0	0	0	0	0	0	
	14	0.78	0.44	0.02	0.86	0.75	1.35	0	0	1.57	0	0	0	0
Community III	Species	1	2	3	4	5	6	7	8	9	10	11	12	
	2	0.86												
	3	0.88	0.31											
	4	0.83	0.41	0.41										
	5	0.63	0.49	0.66	0.26									
	6	0.74	0.97	0.19	0.28	0.60								
	7	0.95	0.27	0.80	0.59	0.41	0.25							
	8	0.53	0.50	0.31	0.42	0.14	0.45	0.50						
	9	1.13	1.06	0.27	0.10	0	0.51	0.12	0.08					
	10	1.15	0.71	0.85	0	0.42	0.11	0.41	0	0.18				
	11	0.41	0.18	0.13	0.53	0.33	0.30	0	0.36	0	0			
	12	0.79	0.41	1.69	0	1.50	0.22	0.82	0	0	0.25	0		
	13	0.23	0.16	0.86	0.03	0.09	0.12	0.09	0.01	0	0	0	0	

Note: Community I: *1 Pinus tabulaeformis; 2: Quercus wutaishansea; 3: Betula platyphylla; 4: Populus davidiana; 5: Swida macrophylla; 6: Lacquer tree; 7: Betula albosinensis; 8: Pyrus betulaefolia; 9: Platycladus orientalis; 10: Cornus controversa; 11: Koelreuteria paniculata; 12: Carya cathayensis; 13: Prunus davidiana;* Community II: *1 Pinus tabulaeformis; 2: Quercus wutaishansea; 3: Lacquer tree; 4: Pyrus betulaefolia; 5: Betula platyphylla; 6: Populus davidiana; 7: Betula albosinensis; 8: Carya cathayensis; 9: Prunus davidiana; 10: Prunus padus; 11: Swida macrophylla; 12: Mountain peach; 13: Acer mono; 14: Cornus controversa;* Community III: *1: Quercus wutaishansea; 2: Pinus tabulaeformis; 3: Populus davidiana; 4: Acer mono; 5: Swida macrophylla; 6: Pyrus betulaefolia; 7: Betula platyphylla; 8: Carya cathayensis; 9: Carpinus turczaninowii; 10: Mountain peach; 11: Prunus davidiana; 12: Betula albosinensis; 13: Lacquer tree*.

Niche overlap was relative high when niche breadths was high, while species was narrow niche breadths resulting in a low potential for overlap. However, there were exceptions. For example, some species with relatively small importance values revealed a wide range of overlap with other species, i.e., the niche overlap between *Cornus controversa*, *Koelreuteria paniculata* and *Betula platyphylla* in community I was 1.129, and niche overlap between *Cornus macrophylla* and *Betula albosinensis* in community III reached 1.501. The values for niche overlap between some species were 0, i.e., *Platycladus orientalis—Carya cathayensis*, *Mountain peach*, *Acer mono—Cornus controversa*, *Betula albosinensis—Lacquer tree*, and others (Table 3). This lack of overlap occurred because the two species were adapted to the habitat in totally different ways; in addition, due to the sparse growth and uneven distribution, the niche between species may overlap in a large habitat range, while it may be separate in small habitat range, resulting in extremely low or 0 values of niche overlap. In brief, according to the changed sequence, values for niche overlaps between heliophilous species (e.g., *Pinus tabulaeformis*) and other populations decreased; niche overlaps between mesophilous species (e.g., *Populus davidiana* and *Acer mono*) and other populations slightly increased; niche overlaps between mesophilous-shade species (e.g., *Quercus wutaishansea*) and other populations significantly increased. Species involved in the latter overlap, with a relatively high abundance, relatively high frequency and dominance, became the dominant species in the community. This process led to the further changes of the forest toward the more stable climax community.

### Overall Association among Dominant Species

The overall interspecies association reflected the overall trend of associations among the species of different changed stages, which was measured by the values of VR. The results showed that the three communities exhibited positive associations for VR > 1 (Table 4); the value of χ^2^ significance test further revealed that the overall associations were significant, i.e., [W > χ^2^_0.05_ (12) = 22.362 and W > χ^2^_0.05_ (13) < W]. In other words, the population of dominant species demonstrated a stable phase and appeared to exist in a mutually beneficial relationship.

**Table 4:** Overall associations among dominant species.

Community types	VR	W	χ^2^ (χ^2^_0.05,N,_χ^2^_0.95,N_)	Results
Community I	1.707	42.685	5.226, 21.026	Positive association, Remarkable
Community II	2.206	55.164	5.892, 22.362	Positive association, Remarkable
Community III	2.612	65.305	5.226, 21.026	Positive association, Remarkable

### Association among Pairs of Main Species

A χ^2^ test can reflect the level of significance of interspecific association accurately and provide a quantitative index for evaluating the significance. In the calculation of the interspecific association coefficient, species with an importance value of at least 1% were included in this study.

Among the total of 45 species pairs composed of 10 dominant species (Table 5) in the *Pinus tabulaeformis* forest (community I), 19 pairs showed positive associations, accounting for 42.22% of the total pairs; 25 pairs showed negative associations, accounting for 55.56% of the total pairs; and 1 pair showed no association. The ratio of positive and negative associations was below 1; positive association referred to a correlation at or above the level of significance, and 1 pair reached a significant correlation, accounting for 2.22% of the total pairs.

**Table 5:** Half matrix of inter-specific association χ^2^ test among dominant species.

Communities	Species	1	2	3	4	5	6	7	8	9
Community I	2	2.50								
	3	0.59	0.03							
	4	0.04	0.20	0.26						
	5	2.00	0.46	0.01	0.06					
	6	0.25	0.01	0.01	0.07	0.02				
	7	0.59	0.03	0.39	0.26	0.01	0.01			
	8	0.90	0.13	0.17	1.50	0.40	0.21	0.17		
	9	1.42	0.35	0.02	2.67	5.00	0.22	0.02	1.00	
	10	5.74	2.50	0.59	0.04	2.00	0.25	0.59	0.90	1.42
Community II	Species	1	2	3	4	5	6	7	8	9
	2	2.50								
	3	1.42	0.35							
	4	0.90	0.13	1.00						
	5	0.25	0.01	0.22	0.21					
	6	0.09	0.11	0.29	5.00	0.00				
	7	0.16	0.05	0.51	0.07	0.50	0.31			
	8	0.25	2.38	0.22	0.21	0.21	0.90	0.50		
	9	0.59	0.03	0.02	0.17	0.01	0.10	0.01	0.01	
	10	0.90	0.13	1.00	0.04	0.57	5.00	0.07	0.21	0.17
Community III	Species	1	2	3	4	5	6	7	8	9
	2	1.42								
	3	0.16	0.37							
	4	0.02	0.05	3.04						
	5	2.00	5.00	1.32	0.03					
	6	0.09	0.55	5.48	0.15	5.53				
	7	0.16	0.37	3.00	0.00	0.09	0.12			
	8	0.59	0.02	0.01	0.50	0.01	0.10	0.01		
	9	0.90	1.00	0.07	0.08	2.98	5.00	0.07	0.17	
	10	1.42	0.07	0.37	2.14	5.00	0.29	0.51	0.02	1.00

Note: Community I: *1: Pinus tabulaeformis, 2: Quercus wutaishansea, 3: Betula platyphylla, 4: Populus davidiana, 5: Swida macrophylla, 6: Lacquer tree, 7: Betula albosinensis, 8: Pyrus betulaefolia, 9: Platycladus orientalis, 10: Cornus controversa;* Community II: 1: *Pinus tabulaeformis, 2: Quercus wutaishansea, 3: Lacquer tree, 4: Pyrus betulaefolia, 5: Betula platyphylla, 6: Populus davidiana, 7: Betula albosinensis, 8: Carya cathayensis, 9: Prunus davidiana, 10: Prunus padu hayensis, 9: Prunus davidiana, 10: Prunus pad;* Community III: *1: Quercus wutaishansea, 2: Pinus tabulaeformis, 3: Populus davidiana, 4: Acer mono, 5: Swida macrophylla, 6: Pyrus betulaefolia, 7: Betula platyphylla, 8: Carya cathayensis, 9: Carpinus turczaninowii Hance, 10: Mountain peach*.

In the *Pinus*+ *Quercus* forest (community II) and the *Quercus wutaishansea* forest (community III) (Table 5), among the 45 species pairs composed of 10 dominant species, 25 pairs showed positive associations, accounting for 55.56% of total pairs; 20 pairs showed negative associations, only accounting for 44.44% of total pairs; and 0 pairs showed no association, and the ratios of positive and negative associations of species were above 1. In community II, 0 pairs reached the level of a significant correlation, and in community III, 2 pairs reached the level of a significant correlation and above, accounting for 4.44% of the total pairs.

### Association Size among Pairs of Dominant Species

**Figure 3:**
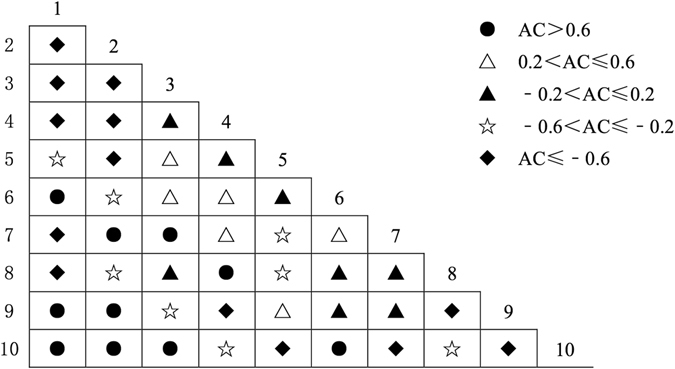
Half-matrix-diagram (AC Value) of interspecific association in Community I. Note: *1: Pinus tabulaeformis, 2: Quercus wutaishansea, 3: Betula platyphylla, 4: Populus davidiana, 5: Swida macrophylla, 6: Lacquer tree, 7: Betula albosinensis, 8: Pyrus betulaefolia, 9: Platycladus orientalis, 10: Cornus controversa*.

**Figure 4:**
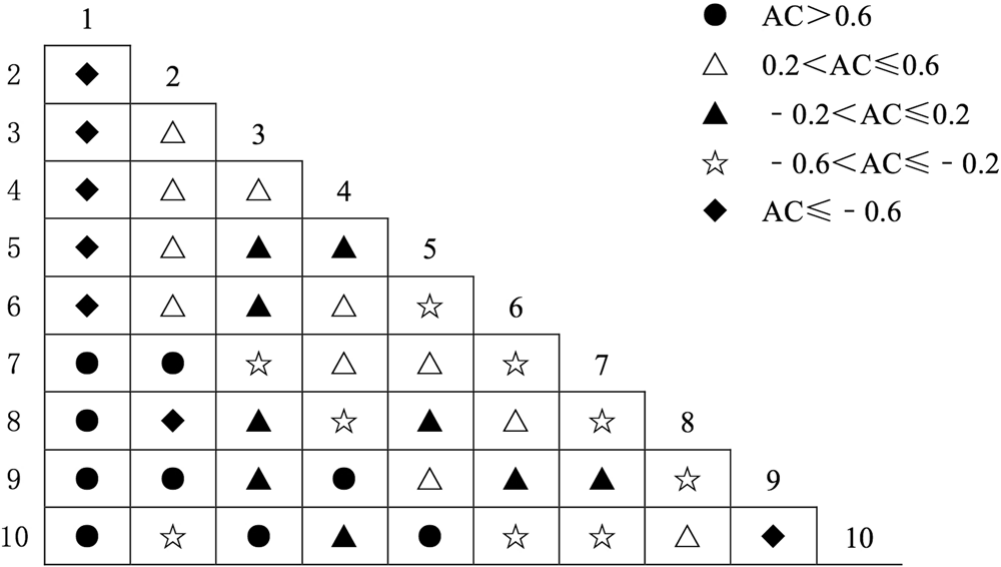
Half-matrix-diagram (AC Value) of interspecific association in Community II. Note: *1: Pinus tabulaeformis, 2: Quercus wutaishansea, 3: Lacquer tree, 4: Pyrus betulaefolia, 5: Betula platyphylla, 6: Populus davidiana, 7: Betula albosinensis, 8: Carya cathayensis, 9: Prunus davidiana, 10: Prunus padus*.

**Figure 5:**
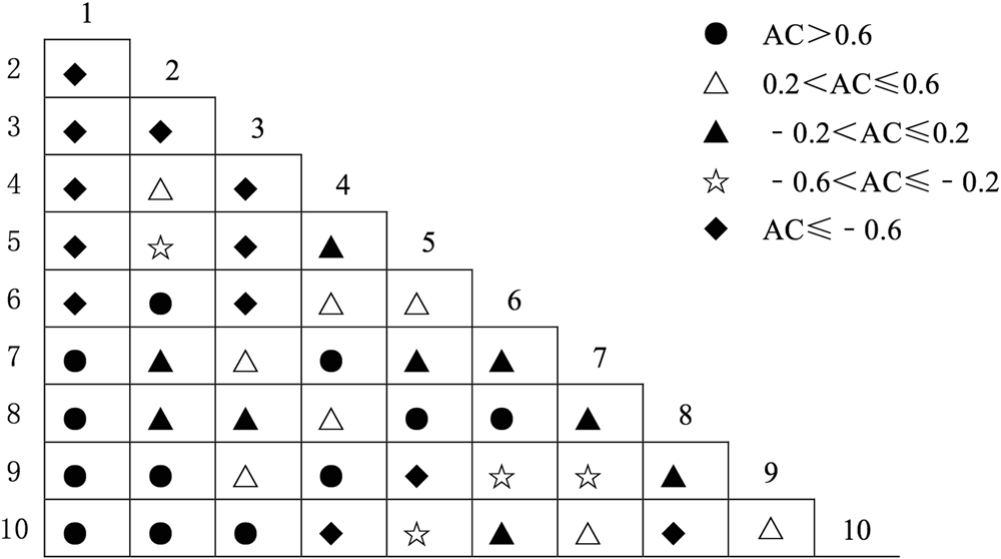
Half-matrix-diagram (AC Value) of interspecific association in Community III. Note: *1: Quercus wutaishansea, 2: Pinus tabulaeformis, 3: Populus davidiana, 4: Acer mono, 5: Swida macrophylla, 6: Pyrus betulaefolia, 7: Betula platyphylla, 8: Carya cathayensis, 9: Carpinus turczaninowii Hance, 10: Mountain peach*.

The Spearman rank correlation coefficient was used to verify the applicability of AC, and the results indicated that using *AC* to represent the interspecific association was reasonable. The semi-matrix diagram of the association coefficient (AC) showed that there were 13 pairs with significant negative associations in community I (Fig. 3), with AC values all equal to −1; 8 pairs with weak negative associations; 8 pairs without association; 6 pairs with weak positive associations; and 10 pairs with significant positive associations. In community II (Fig. 4), there were 7 pairs with significant negative associations, 9 pairs with weak negative associations, 9 pairs without association, 11 pairs with weak positive associations and 9 pairs with significant positive associations. In community III (Fig. 5), there were 12 pairs with significant negative associations, 4 pairs with weak negative associations, 9 pairs without association, 8 pairs with weak positive associations and 12 pairs with significant positive associations.

## Discussions

A community is formed by the continual changes of species, and those species are fitter for the environmental conditions, and also the components of the community^31^. With regard to the natural secondary forest changes on the Loess Plateau, artificial episodic changes (i.e., forest tending) and destruction created production ecology, which enabled heliophilous species to grow well and occupy the dominant position^32^. Research on niche breadth and overlap of the main tree populations in the late changed stage of the natural secondary forest provided additional data regarding the resource utilization. Niche overlap generally occurred among the main species, but it occurred to a lesser extent where the values of niche overlap were 0, which was found for of 17, 22 and 12 species pairs from community I, community II and community III, respectively. This result indicated the structural complexity and the resource diversity due to long-term competition and adaption among populations in different communities.

In the current study, the research area was in the center of the *Quercus wutaishansea* distribution, and environmental conditions such as climate and soil were very suitable for the regeneration and survival. Therefore, in community III, the niche breadth of *Quercus wutaishansea* exceeded that of *Pinus tabulaeformis* and reached a maximum. *Betula platyphylla* and *Populus davidiana*, the heliophilous pioneer species, although they lacked seedlings or saplings and had low abundance, and still had certain niche breadths, which mainly due to their large individual forms and large influence on neighboring species. *Lacquer tree* and *Swida macrophylla*, as slightly heliophilous, were adaptable to the shady, cold and damp forest environment below the high canopy density of the natural broadleaf-conifer secondary forest, so they could distributed in almost all types of communities, with a strong ability for habitat utilization and a relatively large niche breadth. *Carpinus turczaninowii*, *Populus davidiana*, *Mountain peach* and *Carya cathayensis* occupied partial resource niches and distributed unevenly, thus resulting in their relatively small niche breadth, and reflected their relatively weak ability to utilize and adapt environmental resources. Thus, these species tended to be distributed in specialized habitats and only existed as co-dominant species or companion species of dominant species in some types of communities.

*Quercus wutaishansea* was a mesophytic tolerant tree of cold and drought, which made it more fit for growing under trees, and the *Pinus tabulaeformis* forest could create conditions for the settlement of *Quercus wutaishansea* seedlings and would gradually develop into a *Pinus*+ *Quercus* mixed forest (community II). Finally, a mixed forest with dominant *Quercus wutaishansea* or a pure *Quercus wutaishansea* forest would develop (community III). This process was consistent with the results of research on nearly 50 years of vegetation change and development on the northern part of Ziwu Mountains^11^. Conversely, as *Pinus tabulaeformis* seedlings were not tolerant of low light supplies, and could not invade a climax community; therefore, the *Quercus wutaishansea* forest would not form a mixed forest or even a pure *Pinus tabulaeformis* forest.

Patterns of association changed, which may be due in part to changes in nutrient gradients^4, 27^. Initially, a few successful species may compete for the same resources, while later, many species partition different resource patches or parts of gradients^7, 33^. Therefore, the strength of interaction would decrease with time, as was shown in the decline during changes in the number of positive and negative species pairs^34^. The association pattern and experiments had demonstrated a closer correspondence, that competitive interactions had more significance than allelopathic interactions^35, 36^. However, competition may be easier to establish than allelopathy because allelopathic mechanisms may be specific to both site and species^6^.

The dominant species with an overall positive association (VR > 1) illustrated that the communities were relatively stable. In general, the stability of the community structure and species composition was enhanced by the community changed process^37^. The closer the community was to the climax stage, the greater the ecological stability^38, 39^. The species of *Pinus tabulaeformis* and *Quercus wutaishansea* were important components of the evergreen broadleaf forest and temperate coniferous forests in China. Therefore, we can conclude that the studied forest on the Loess Plateau could share unlimited resources and were at the stage of late changes or in a relatively dynamic balance. Species pairs with positive associations indicated that the plants could shared similar resources and exhibited a wide niche overlap. For instance, *Betula platyphylla* and *Populus davidiana* had positive associations, which suggested that the breadth and overlap of the regeneration niche were both relatively large. Our findings were consistent with the results presented by Gong ZW^40^. In contrast, a negative association indicated that these plants had different environmental requirements^41^. For example, *Pinus tabulaeformis* can create a regeneration environment, which was suitable for the species of *Quercus wutaishansea*, which had difficulty regenerating in intense light conditions.

In this changed series, the ratio of positive to negative associations in the *Pinus tabulaeformis* community was below 1, indicating a possible negative correlation among the majority of the species pairs, but the significance test showed that most species were in positive association, and an analysis on multi-species association showed that all tree species in each community had significant associations on the whole. Some researchers considered that the ratios of positive to negative associations were below 1 may be caused by the secondary nature and youth of the communities and artificial episodic changes^42^. This suggestion implied that secondary, young and artificially disturbed communities may have more negative interspecies associations. In 1998, “Natural Forest Protection Project” was implemented in this research area, and artificial cutting and destruction had been effectively restrained. Before implementation, the forest vegetation on the Loess Plateau had been undergoing severe artificial cutting and disturbance, and the community had shown an obvious secondary structure, and would this historical condition cause the ratios of positive and negative associations, which required further investigation.

The comparison of the communities revealed that the *Quercus wutaishansea* forest in the climax stage exhibited the weakest interspecies association, and the number of species pairs with significant interspecies association decreased, with most of them showing positive associations. In the early stage of changes (Community I), species had close interspecies relationships during the processes of immigration, ecesis, aggregation, competition and reaction, whereas these interspecies associations decreased in the middle and late changed stages (Community II and III). Therefore, the *Quercus wutaishansea* forest (Community III) was a climax community, and it was already in the stable state of a natural climax community, and the invasive species were basically mesophytic species, which had similar adaptability to the environment, the interspecies associations were mostly positive. Myster and Pickett considered that the proportion of species pairs in positive association increased over the course of a time series^43^, and correspondingly, the number of without association gradually increased and the proportion of negative association tended to decrease, which was fully consistent with our research^44^. Namely, with the progression of changes and the increasingly high stability of the community, associations between multiple species may gradually involve positively^45, 46^.

Although many species were present on the Loess Plateau, our results showed that the interspecific association with an extremely significant level was low, and the results were consistent with the findings in the subtropical evergreen broadleaf forest^24^. The dominance of associated species, especially in community I, agreed well with studies at other sites^42^. Many of these species were also important in defining changed pathways. From community I to community III, secondary change was characterized by the variation in abundance of *Betula platyphylla* and *Populus davidiana*.

The associations and niches overlap reflected the difference in community habitat, and thus, the significance of interspecies association necessarily had a certain relationship with community stability^47^. This correlation was consistent with the status as dominant species in the communities of changed series^48^, because the status of dominant species had been established through a series of interspecies interactions, and its final change may be subject to the change of community habitat and relatively weakly affected by other species^49, 50^. Therefore, communities in later changed stages (Dominated by the species of *Quercus wutaishansea*) were stable, which contrast to the other communities. In the study area, the analysis of interspecies associations was basically consistent with that of niche, and species pairs with high positive associations had high niche overlap, reflecting these species pairs’ similarity in habitat requirements. For the restoration and rebuilding of degraded ecosystems, especially the restoration of a complex ecosystem with high species diversity, consideration should be given to the population structure and niche differentiation of species. The role of plants in the restoration and rebuilding of ecosystems can be explained by applying niche theory.
